# Differing Spontaneous Brain Activity in Healthy Adults with Two Different Body Constitutions: A Resting-State Functional Magnetic Resonance Imaging Study

**DOI:** 10.3390/jcm8070951

**Published:** 2019-06-30

**Authors:** Ching-Hsiung Liu, Yen-Ying Kung, Tzu-Chen Yeh, Pei-Shan Hsu, Ching-Ju Yang, Chou-Ming Cheng, Hong-Chun Lin, Jen-Lin Yang, Ta-Peng Wu, Ching-Mao Chang, Jen-Chuen Hsieh, Fang-Pey Chen

**Affiliations:** 1 Department of Neurology, Lotung Poh-Ai Hospital, Ilan 26514, Taiwan; 2 Institute of Traditional Medicine, School of Medicine, National Yang-Ming University, Taipei 11221, Taiwan; 3 Integrated Brain Research Unit, Department of Medical Research, Taipei Veterans General Hospital, Taipei 11267, Taiwan; 4 Center for Traditional Medicine, Taipei Veterans General Hospital, Taipei 11267, Taiwan; 5 Institute of Brain Science, School of Medicine, National Yang-Ming University, Taipei 11221, Taiwan; 6 Department of Chinese Medicine, Taipei Tzu Chi Hospital, Buddhist Tzu Chi Medical Foundation, New Taipei City 23142, Taiwan; 7 Faculty of Medicine, School of Medicine, Yang-Ming University, Taipei 11221, Taiwan; 8 Department of Radiology, Taipei Veterans General Hospital, Taipei 11267, Taiwan

**Keywords:** body constitution, magnetic resonance imaging, quality of life, traditional Chinese medicine

## Abstract

Traditional Chinese medicine (TCM) practitioners assess body constitution (BC) as a treatment basis for maintaining body homeostasis. We investigated patterns in spontaneous brain activity in different BC groups using resting-state functional magnetic resonance imaging (rsfMRI) and determined the relationship between these patterns and quality of life (QOL). Thirty-two healthy individuals divided into two groups (body constitution questionnaire (BCQ)-gentleness [BCQ-G] and BCQ-deficiency [BCQ-D]) based on the body constitution questionnaire (BCQ) underwent rsfMRI to analyze regional homogeneity (ReHo) and the amplitude of low-frequency fluctuation (ALFF). The World Health Organization Quality of Life Instruments (brief edition) scale was used to evaluate the QOL. The BCQ-G group (*n* = 18) had significantly greater ReHo values in the right postcentral gyrus and lower ALFF values in the brainstem than the BCQ-D group (*n* = 14). In the BCQ-D group, decreased ReHo of the postcentral gyrus correlated with better physiological functioning; increased ALFF in the brainstem correlated with poor QOL. BCQ-subgroup analysis revealed a nonsignificant correlation between ReHo and Yang deficiency/phlegm and stasis (Phl & STA). Nonetheless, the BCQ-D group showed a positive correlation between ALFF and Phl & STA in the parahippocampus. This study identified differences between BCQ-G and BCQ-D types of healthy adults based on the rsfMRI analysis. The different BCQ types with varied brain endophenotypes may elucidate individualized TCM treatment strategies.

## 1. Introduction

Traditional Chinese medicine (TCM) has been practiced in China for 2000 years. TCM practitioners assess individual innate characters as patterns of body constitution (BC) according to the yin-yang theory and treat patients with herbal medicine, tuina, or acupuncture in clinical practice. TCM treats the mind–body as a holistic system [1,2,3]. Based on TCM theory, diseases originate from an unbalance of yin and yang in the mind–body and the attenuation of BC [3,4]. 

The most widely used instruments for BC classification were developed by Wang and Su. Wang et al. established a constitutional rule of nine that forms the primary content of the ‘Constitution in Chinese Medicine Questionnaire’ (CCMQ). This includes gentleness, qi-deficiency, yang-deficiency, yin-deficiency, phlegm-dampness, dampness-heat, blood-stasis, qi-depression, and special diathesis [5,6]. Su et al. [3,4] created a 42-item questionnaire for assessing BC (body constitution questionnaire [BCQ]), which includes healthy/sub-healthy conditions consisting of gentleness, yang-deficiency (YaD), yin-deficiency (YinD), and phlegm and stasis (Phl & STA). YaD is characterized by cold intolerance, which may correlate with dysfunction of the hypothalamic–pituitary–adrenal axis [7]; YinD is characterized by heat intolerance, which may correlate with lipid metabolic gene regulation [7,8]. Phl & STA is associated with blood lipid and sugar metabolism [9]. In TCM practice, an individual’s BC represents the constitutional traits of an individual’s morphosis, including physical and psychological components that determine susceptibility to pathogenic factors [1,10].

Functional magnetic resonance imaging (fMRI) is a useful tool that can link the brain (endophenotype) and behavior (phenotype), including physiological phenomena or pathological disorders of the central nervous system [11,12]. This imaging approach is based on assessing blood oxygen level-dependent (BOLD) signal changes as markers of brain activity changes. Two methods can be used to measure different aspects of brain–behavior interactions: Task-based or resting-state fMRI (rsfMRI) [13,14]. Recently, rsfMRI studies have enabled further understanding of human brain plasticity [15,16]. Recent rsfMRI studies have assessed the neural mechanisms of TCM, such as acupuncture treatment, in various diseases [17,18]. However, few fMRI studies have assessed the TCM theory [19,20]. Differential resting-state brain activity has been detected in patients with yin and yang-type depression [20]. Therefore, neural activity measures may be suited for tracing the early brain function properties of different BCQ groups in healthy adults.

Regional homogeneity (ReHo) and amplitude of low-frequency fluctuation (ALFF) analyses are two vital methods for measuring local neural activity in the brain [21,22]. ReHo analysis is calculated from the direct neighborhood of single voxels for measuring the time-series similarity of its nearest neighbors [23]. ALFF estimates the total power of the BOLD signal within a low-frequency range (0.01–0.08 Hz) [22,24]. Previous studies have indicated that ReHo and ALFF show alternations with similar distributions in different pathological conditions [25,26]. However, ReHo may be more sensitive than ALFF at detecting regional abnormalities, and ALFF may be more effective at measuring global spontaneous activity [27]. Consequently, the combination of the two analyses may provide more information on regional brain spontaneous activity than either alone [22,27]. 

In the present study, we used ReHo and ALFF analyses to inspect global spontaneous neuronal activity in the brain of healthy adults with different BCQ types. We hypothesized that different BCQ types of healthy adults would have different ReHo and ALFF maps related to brain regions. Further, changes of the spontaneous brain activity would be related to the behavior scales and the sub-patterns of BCQ-related measurements.

## 2. Methods

### 2.1. Study Design and Participants

This study was approved by the institutional review board of the Taipei Veterans General Hospital. All participants provided written informed consent. We recruited 35 healthy individuals from the local community via advertisements. Inclusion criteria were: Age 35–70 years; no organic brain disorder; no current use of medications for chronic illness; and no history of any psychiatric/neurological disease or surgery. All participants completed the BCQ and World Health Organization Quality of Life Instruments (brief edition) (WHOQOL-BREF) questionnaires before undergoing rsfMRI. The WHOQOL-BREF has four subdomains: (1) Physical domain (score range, 4–20); (2) psychological domain (score range, 4–20); (3) social domain (score range, 4–20); and (4) environmental domain (score range, 4–20). The total score ranges 8–80 points, with higher values indicating better QOL [28]. 

Three individuals were excluded from this study because of claustrophobia during the MRI examination (*n* = 1) or organic brain lesions, revealed by MRI (*n* = 2). Thus, finally, we included 32 healthy individuals and divided them into two groups based on the BCQ. BCQ-gentleness (BCQ-G) type was defined as individuals with no YaD, YinD, or Phl & STA. BCQ-deficiency (BCQ-D) type was defined as individuals with YaD or YinD. The BCQ contains three subtypes: YaD (score range, 19–95), YinD (score range, 19–95), and Phl & STA (score range, 16–80); the cut-off points for the scores of YinD, YaD, and Phl & STA are ≥ 30 points, 31 points, and 27 points, respectively.

A flowchart of the study design is shown in Supplementary Figure S1. 

### 2.2. MRI Acquisition

fMRI was performed with a 3.0 Tesla scanner (Discovery MR750, GE Healthcare, Waukesha, WI, USA) at the Integrated Brain Research Unit, Department of Medical Research & Education, Taipei Veterans General Hospital, Taipei, Taiwan. A 12-channel head coil was used, along with restraining vacuum pads to minimize head motion and to reduce scanner noise. Sequence of gradient echo-planar image (EPI) was used for the rsfMRI scan with the following parameters: Repetition number = 200 for conventional EPI; dummy scan, 5; TR (repetition time)/TE (echo time) = 2000/30 ms; flip angle = 90°; matrix size = 64 × 64 × 40; slice number = 40; slice thickness = 3 mm; and field of view (FOV) = 240 × 240 mm^2^. 

The baseline resting brain activity was acquired as follows: (1) The first five EPI scans of each rsfMRI series were discarded for signal saturation and magnetic-field stabilization; and (2) during the 7-min fMRI scanning, individuals were instructed to keep their eyes closed, relax, move as little as possible, and stay awake, for which they were asked to hold a plastic ball. An online real-time analysis of head motion using the methods modified from Analysis of Functional Neuroimaging (NIMH, Bethesda, MD, USA) was performed to confirm the quality of rsfMRI images, with a head translation of 1 mm and head rotation < 0.5° for each session. If the head motion exceeded the motion criteria, data were excluded from the analysis. Structural images were acquired using a three-dimensional T1-weighted structural image (TR/TE/TI [inversion time] = 8.2/3.2/450 ms; flip angle = 12°; matrix size = 256 × 256 × 176; FOV = 230 × 230 mm^2^; and voxel size = 0.9 × 0.9 × 0.9 mm^3^). The total rsfMRI scan time was approximately 11 min. Head cushions and earplugs were provided to reduce head motion and noise during the scans.

### 2.3. Data Preprocessing

fMRI data preprocessing was performed using the toolbox Data Processing Assistant for Resting-state fMRI V4.4 advanced edition (DPARSF-A, Key Laboratory of Behavioral Science and Magnetic Resonance Imaging Research Center, Institute of Psychology, Chinese Academy of Sciences) [29] with Statistical Parametrical Mapping 12 (SPM; https://www.fil.ion.ucl.ac.uk/spm/software/spm12) in MATLAB 2014a (The Math Works, Inc., Natick, MA, USA). Functional image data were corrected for any head movements using SPM-realign, a linear transformation procedure. We coregistered structural data with SPM-coregister and normalized to Montreal Neurological Institute (MNI) space using a standard MNI template (SPM-normalize). Next, the data were smoothed with SPM-smooth. During the fMRI examination, the participant’s vital signs, such as heart rate, oxygen saturation, and respiratory rate, were monitored and recorded.

### 2.4. ALFF and ReHo Analyses

ALFF and ReHo analyses were performed based on previous studies [23,24] using the Data Processing & Analysis for Resting-State Brain Imaging (DPABI) [29]. For ALFF analysis, the resampled functional images were smoothed with a 3D Gaussian kernel of 4 mm full-width at half-maximum (FWHM). De-trend and band-pass filtering (0.01–0.08 Hz) were performed to remove the effects of low-frequency drift and high-frequency noise. The following nuisance variables were regressed out: (1) Six head movement parameters computed based on rigid body translation SPM12; (2) mean signal within the lateral ventricles of cerebrospinal fluid; and (3) mean signal within a deep white matter region (centrum ovale). Subsequently, the time series were transformed into the frequency domain using a Fourier transform. Next, the square root of the power spectrum was calculated and averaged across 0.01–0.08 Hz within each voxel to obtain a raw ALFF map. Following this, the global mean ALFF value was calculated by extracting and averaging the raw values from all voxels across the whole brain. Finally, ALFF values for each voxel were divided by the global mean ALFF value for standardization. The resulting ALFF value in a given voxel reflected the degree of its raw ALFF value relative to the average ALFF value of the whole brain [24].

ReHo analysis was performed on the functional images after preprocessing. The nuisance variables were regressed out using the same methods for calculating the ALFF value. Briefly, after de-trend and band-pass filtering were performed, ReHo maps were produced by calculating the concordance of the Kendall coefficient of the time series of a given voxel with its 26 nearest neighbors [23]. Next, the ReHo value of each voxel was standardized by dividing the raw value by the global mean ReHo value. Finally, ReHo data were smoothed using a 3D Gaussian kernel of 4 mm FWHM for further statistical analysis.

### 2.5. Statistical Analysis

Demographic and behavioral data were compared with the χ^2^ test (categorical variables) and independent *t*-test (continuous variables) using SPSS (Version 19.0, SPSS Inc., Chicago, IL, USA). Pearson’s correlation analysis was applied to assess the degree of association between two variables. Results were considered statistically significant if *p* < 0.05.

#### 2.5.1. Within-Group Analysis

One-sample *t*-tests were performed on the ALFF and ReHo maps for each group to display the most significant results and reflect the intrinsic characters of these two groups. A conservative statistical significance was set at voxel level *p* < 0.001 and cluster size > 150 voxels, which corresponded to a multiple comparisons correction with family-wise error rate (FWE) of *p* < 0.05.

#### 2.5.2. Between-Group Analysis

Independent two-sample *t*-tests were performed with a gray matter mask to investigate the between-group differences of ALFF and ReHo values. Global signal regression was imported, and age and sex were included as covariates. The statistical voxel threshold was set at *p* < 0.005 and a cluster size > 35 voxels, which corresponded to an FWE correction of *p* < 0.05.

#### 2.5.3. Correlation Analysis

We performed a correlation analysis to investigate the association between ALFF/ReHo values and the behavior scales (WHOQOL-BREF and BCQ). WHOQOL-BREF and its subdomains were used to assess multiple factors of QOL associated with adaptive or maladaptive changes in the brain region. To explore the most significant correlations among the ALFF/ReHo MRI values, the statistical threshold was set at voxel level *p* < 0.005 and cluster size > 35 voxels, which corresponded to a *p* < 0.05 (FWE-corrected). 

## 3. Results

### 3.1. Demographic Data

A total of 32 participants were included in the data analysis. The BCQ-G and BCQ-D groups did not differ statistically in terms of age, sex, body mass index, and scores of WHOQOL-BREF. However, there were significant differences in BCQ-subtype between the groups (*p* < 0.001; Table 1).

### 3.2. Behavior Scale Correlations in the Different Groups

YaD and Phl & STA scores were negatively correlated with several variables, such as the psychological domain and sum of the WHOQOL-BREF scores. However, there was no significant correlation between BCQ subscores and WHOQOL-BREF when the groups were divided into BCQ subtypes (Table 2).

### 3.3. ReHo and ALFF Analyses

**Figure 1:**
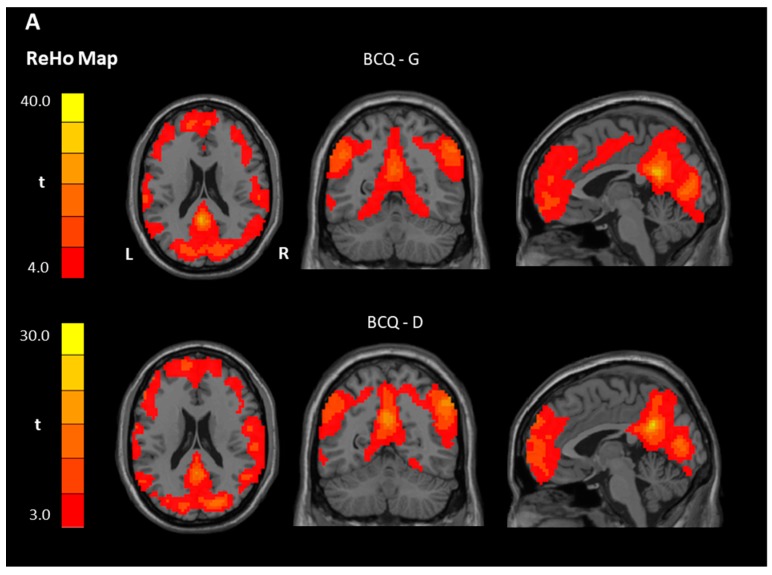

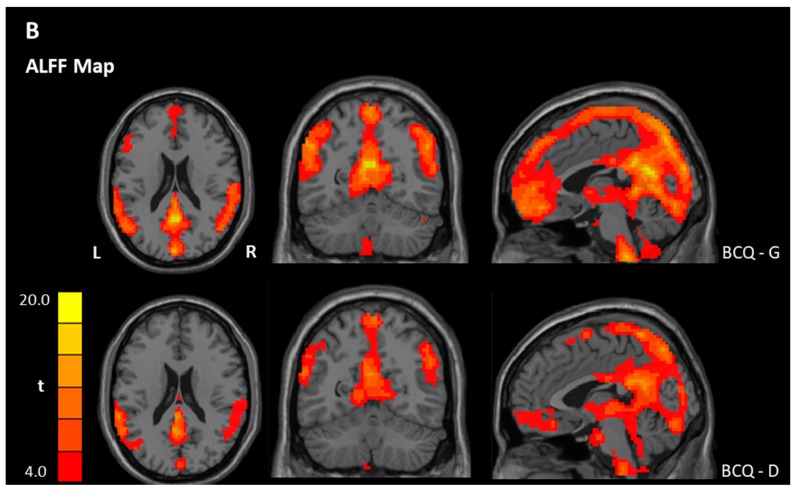
Representative one-sample *t*-test results of ReHo and ALFF maps (*p* < 0.005, family-wise error rate (FWE) corrected). Within each group, standardized ReHo (**A**) and ALFF (**B**) values in the PCC and the medial PFC were significantly greater than the global mean values in both groups. Other regions, such as the inferior parietal lobe and bilateral occipital lobes, also had greater spontaneous activity. Note that the brain regions were mainly present in the default-mode network, which was not significantly different between the groups in both analyses. ReHo, regional homogeneity; ALFF, amplitude of low-frequency fluctuation; R, right; L, left; BCQ-G, gentleness type of body constitution questionnaire; BCQ-D, deficiency type of body constitution questionnaire; FWE, family-wise error rate; PCC, posterior cingulate cortex; PFC, prefrontal cortex.

**Figure 2:**
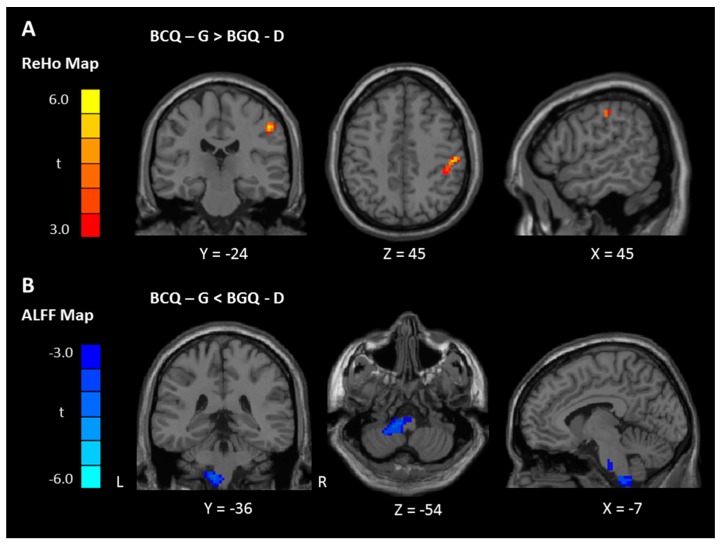
ReHo (**A**) and ALFF (**B**) differences between BCQ-G and BCQ-D healthy subjects (*p* < 0.05, FWE corrected). **A**: Compared with BCQ-D subjects, Subjects with significantly increased ReHo in the postcentral gyrus (red). **B**: Compared with BCQ-D subjects, BCQ-G type showed significantly decreased ALFF in the brainstem (pons) and cerebellum (blue). ReHo, regional homogeneity; ALFF, amplitude of low-frequency fluctuation; BCQ-G, gentleness type of body constitution questionnaire; BCQ-D, deficiency type of body constitution questionnaire; FWE, family-wise error rate; color scale denotes the t value. x, y, z, Montreal Neurological Institute coordinates; R, right; L, left.

In the BCQ-G group, both ReHo and ALFF signals were significantly higher than the global mean values in the posterior cingulate cortex (PCC). In addition, no region showed a lower signal than the average values in both analyses. In the BCQ-D group, the orbitofrontal cortex (OFC) showed higher ReHo and ALFF values than the global mean values. Further, there were higher ReHo values than the global average values in the PCC. Interestingly, the BCQ-D group revealed lower ALFF values than the global mean value in the precentral gyrus (Table 3 and Figure 1). Finally, BCQ-G group values were significantly increased in the postcentral gyrus (ReHo) and decreased in the left brainstem/cerebellum (ALFF) when compared with the BCG-D group (Table 3 and Figure 2A,B).

### 3.4. Correlation Analysis

**Figure 3:**
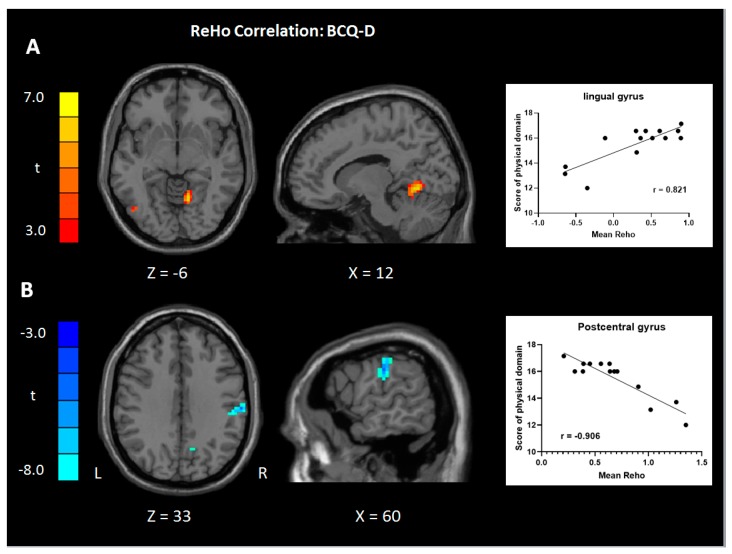

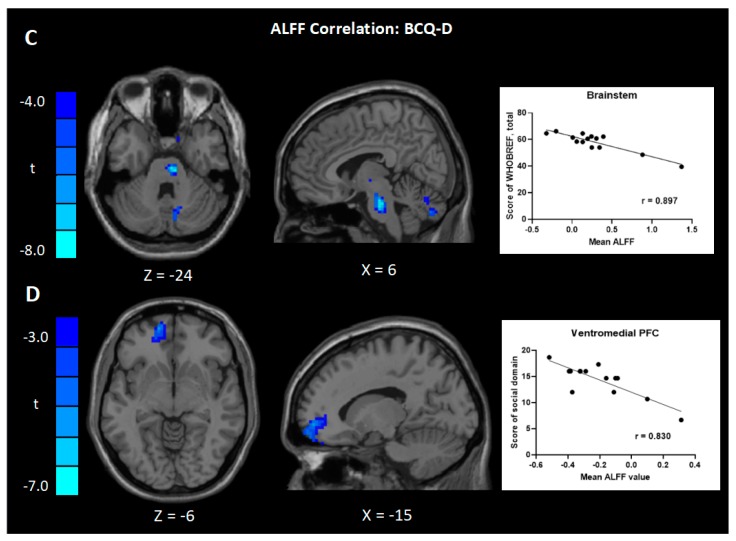
Correlations between the ReHo (**A**,**B**) and ALFF (**C**,**D**) values extracted from significantly different regions of BCQ-D subjects (*p* < 0.05, FWE corrected). The mean ReHo values had a significantly positive correlation between the physical domain of WHOQOL-BREF and the region in the right lingual gyrus (**A**, *r* = 0.821, *p* = 0.0003), and negative correlation between the physical domain of WHOQOL-BREF and the region in the right posterior central gyrus (**B**; *r* = –0.906, *p* < 0.0001). The ALFF values had a significantly negative correlation between the total scores of WHOQOL-BREF and the region in the right brainstem (**C**; *r* = –0.897, *p* = 0.0001), and a significantly negative correlation between the social domain of WHOQOL-BREF and the region in the left ventromedial prefrontal cortex. (**D**; *r* = 0.830, *p* = 0.0002). ReHo, regional homogeneity; ALFF, amplitude of low-frequency fluctuation; PFC, prefrontal cortex; WHOQOL-BREF, World Health Organization Quality of Life Instruments (brief edition); BCQ-D, deficiency type of body constitution questionnaire; FWE, family-wise error rate; color scale denotes the *t* value; x, y, z, Montreal Neurological Institute coordinates; R, right; L, left.

Increased ReHo values in the cerebellum/lingual gyrus were positively correlated with the physical subdomains of WHOQOL-BREF in the BCQ-D group when compared with the BCQ-G group (Figure 3A). There were no significant correlations between ReHo/ALFF values and WHOQO-BREF and its subdomains in the BCQ-G group. Besides, in the BCQ-D group, there were significant correlations between increased neural activity (ReHo/ALFF) in the postcentral gyrus, brainstem, and negative association with ventromedial prefrontal cortex (vmPFC) and physical-social performance, as measured by the total WHOQOL-BREF scores and subdomains (Table 4 and Figure 3B–D). 

Next, we pooled all participants into one group to assess the correlation between BCQ subtypes (YinD, Yang D, and Phl & STA) and ALFF/ReHo values after regressing out the variables of age and sex. There were no significant clusters survived after FWE correction. However, in ALFF analysis, the parahippocampus (Parahippo1, BA 36), OFC, and Parahippo2 (BA 36) were significantly positively correlated with ALFF values in the YaD and Phl & STA groups (Table 5 and Figure S2). 

**Figure 4:**
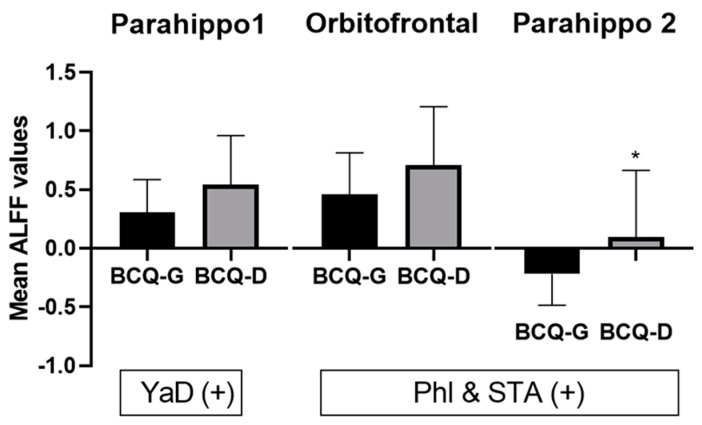
Differences in ALFF values between the groups. The mean ALFF values extracted from the related brain regions (Parahippo1 [BA 36], orbitofrontal cortex, and Parahippo2 [BA 36], which were positively correlated to YaD and Phl & STA, respectively) nevertheless, showed increased values than those in the BCQ-D group, and significant differences in the region of Parahippo2 between BCQ-G and BCQ-D groups. * *p* < 0.05; ReHo, regional homogeneity; ALFF, amplitude of low-frequency fluctuation; BCQ, body constitution questionnaire; SMA, supplementary motor area; Parahippo, parahippocampus; BA, Brodmann area; YinD, yin-deficiency; YaD, yang-deficiency; Phl & STA, phlegm and stasis.

Further, we extracted ALFF values from these regions showing significant correlations, which revealed that the BCQ-D group showed increased ALFF values in the Parahippo1, Parahippo2, and OFC regions (Figure 4). In addition, the BCQ-D group has a significant positive correlation between ALFF values and YaD and Phl & STA scores in the parahippocampus and OFC, respectively (Figure 4; Table 5).

## 4. Discussion

To the best of our knowledge, this is the first study to use rsfMRI to evaluate BC according to TCM theory. We have explored the changes in spontaneous brain activity in healthy adults with two types of BC, BCQ-G, and BCQ-D. Compared with the BCQ-G group, the BCQ-D group had decreased ReHo values in the right postcentral gyrus (associated with the primary somatosensory cortex). Further, the BCQ-D group had increased ALFF values in the left pons/cerebellum.

Decreased ReHo in the right somatosensory cortex of BCQ-D individuals was associated with WHOQOL-BREF scores for better physiological functions, which meant an adaptive alternation of ReHo in these individuals. However, increased ALFF in the brainstem of BCQ-D individuals was associated with lower total WHOQOL-BREF scores, which meant a maladaptive alteration of ALFF in these individuals. In addition, the BCQ subtype analysis (*n* = 32) found that increased ALFF in the parahippocampus and OFC was associated with more points in YaD and Phl & STA subtypes. Nevertheless, there was a nonsignificant correlation between ReHo and BCQ-subtype. Our results suggested ReHo values in the primary somatosensory cortex may contribute to the homeostasis in both BCQ type and increased ALFF in the parahippocampus may play a role in deficiency-type of BC. Besides, ALFF analysis may exhibit an attenuation of neural activity but may not be seen in ReHo. 

The major findings of this study show that BCQ-D individuals had significantly decreased ReHo values in the postcentral gyrus and increased ALFF values in the pons/cerebellum (peduncle). The postcentral gyrus corresponds to the primary somatosensory cortex (BA 3, 1, 2), which is associated with conscious perception of pain (location, intensity, and noxious stimulation), somatic sensation, and proprioception [30,31]. The primary somatosensory cortex plays a significant role in processing afferent somatosensory input and contributes to integrating the sensory and motor signals for necessary movement [32]. The sub-items of BCQ questionnaire about YinD including the hot sensation over chest/palm/sole area, tinnitus, and dry mouths and lips; and YaD including the sudden blackout in vision, cold intolerance/cold limbs and weakness/coldness/ache of waist, knee and heels and vertigo [33,34]. Therefore, comparing to BCQ-G group, the BCQ-D exhibited decreased ReHo in the postcentral gyrus which may pertain to the alteration in the primary sensory modalities involving body sensation (e.g., vestibular and somatosensation) and from external environment stimulation (e.g., hearing and vision) [32,35].

Previous fMRI studies on the functional disorder or pre-disease state have reported increased ReHo activity in the postcentral gyrus associated with irritable bowel syndrome (IBS) with depression and mild cognitive impairment (MCI) respectively [36,37]. Moreover, experimentally induced low back pain in healthy subjects showed decreased ReHo in the primary somatosensory cortex after painful stimulation [38]. Therefore, altered regional homogeneity in the postcentral gyrus may contribute to pain procession and emotional dysfunction of the sensorimotor network. Besides, recent studies revealed that a deficiency in yin and yang is a frequent syndrome pattern in Parkinson disease (PD) and diabetes [39,40]. These reports suggest that the deficiency syndrome pattern may correlate with neuronal pathophysiology. Our result of BCQ-D individuals showing decreased ReHo in the postcentral gyrus may imply that BCQ-D group may have potential alterations in multi-networks of pain processing, movement coherence, and systemic metabolic arrangement. 

Additionally, the BCQ-D individuals showed significant ALFF increases in the left brainstem(pons)/cerebellum, which was also reported by previous studies in migraineurs (pons) and patients with social anxiety disorder (cerebellum) [41,42]. Pain transmission is linked to the brainstem and associated with the descending pain modulatory system [43]. The cerebellum is in charge of motor control and coordination; and also engaging in pain and emotional processing [44,45]. However, the association between BCQ-D and potential pain-perception disorder or neuropsychiatric abnormality remains unknown.

The correlation analysis revealed that increased ReHo coherence in the cerebellum (lingual)/lingual gyrus and postcentral gyrus was associated with increased/decreased physiological scores in the WHOQOL-BREF subdomain in the BCQ-D group. The anterior lobe of the cerebellum (lingula) receives vestibular connections from the brainstem, mediates body posture, and may connect to the primary somatomotor cortex [46,47]. Increased ReHo in the right cerebellum is associated with increased visual organizational skills [48]. Additionally, neural coherence decreased in the postcentral gyrus of BCQ-D individuals, which was negatively correlated to the physiological subdomain of the WHOQOL-BREF. These results are consistent with previous findings of ALFF of increasing ReHo in the postcentral gyrus may relate to healthy adults with the functional disorder of IBS with depression or MCI [36,37], and decreased ReHo in the postcentral gyrus may associate with better physiological function in our study of BCQ-D group. This suggests that increased local coherence in the cerebellum/lingual gyrus and decreased ReHo in the postcentral gyrus may contribute to functional integration between cerebellar-cortical circuits and somatosensory network to maintain daily physiological activity homeostatically in the BCQ-D group. Besides, increased ALFF values were shown in the brainstem and vmPFC in BCQ-D individuals, which were negatively correlated with total WHOQOL-BREF and social subdomain scores. A previous study assessing lower back pain had shown that increased ALFF in the inferior temporal gyrus was negatively correlated with the activities of daily living of Barthel index for activities of daily living [49], but our results revealed that the brainstem (pons) was a region that reflects QOL. Furthermore, the previous studies linking vmPFC activity to placebo analgesia, fear extinction and generalization which may suggest a general role of vmPFC in inhibiting negative emotion [50,51], yet still have evidence that the vmPFC plays a role in the generation of negative emotion [52,53,54]. Our current result of decreased ALFF in the vmPFC associated with the better social function provided evidence about the vmPFC related to a negative feeling.

In the present study, we used a novel approach utilizing modern rsfMRI with BC analysis, based on TCM. We sought to assess the association between subtypes of BC with brain endophenotypes. Though there were no survival voxels passed under FWE correction in ReHO analysis, the ALFF analysis of correlation with BCQ subtype showed that ALFF values had a significantly positive association with yang-deficiency in the left parahippocampus, and with Phl & STA in the left parahippocampus and OFC. In subgroup analysis, the subscore of Ph1 & STA in BCQ-D group revealed significantly increased ALFF in the parahippocampus than in BCQ-G group. (Figure 4). In TCM theory, YaD constitution was commonly coincident with Phl & STA. The unbalanced BC may exhibit unbalanced condition between qi, blood, yin and yang, frequently determine an individual’s susceptibility to certain pathogenic condition and related diseases [3].

A previous rsfMRI meta-analysis suggested that increased ReHo activity in first-episode drug-naive patients with major depressive disorder (MDD) was predominantly located in the left SMA, left parahippocampal gyrus, and hippocampus [55]. In our study, hyperactivity was found in the left parahippocampus (BA 36), which positively correlated with Phl & STA subscores in the BCQ-D group. Therefore, increased parahippocampal activity may be a possible risk factor of depressive mood that contributes to BC deficiencies, specifically linked to phlegm and stagnation. In TCM, MDD patients were classified as TCM syndrome of deficiency pattern based on the clinical syndrome, pulse, and lingual sign [56]. MDD syndrome is associated with liver stagnation, heart-spleen deficiency, and phlegm misting the mind [57]. However, the relationship between BCQ-D and prevalence of depression remains unclear. Additionally, there were significant positive correlations between ALFF values in the OFC and the Phl & STA subgroup (Figure 4). The OFC is engaged in executive function and behavioral processing that can inhibit neural activity associated with contextually irrelevant, unwanted information (for example, painful sensations and negative emotions) [58]. A previous ALFF study has shown that cognitive vulnerability to depression was associated with reduced ALFF values in the bilateral OFC [59]. In our study, neural activity in the OFC was positively correlated with Phl & STA group scores, indicating that individuals in the BCQ-D group may activate the OFC to compensate for negative information and maintain body–mind homeostasis. 

ReHo and ALFF are the majority of analytic techniques for rsfMRI data that address low frequency fluctuations. The former is computed only from the direct neighborhood of single voxels, and the latter calculates the voxel-wise magnitude of specific frequency bands in the frequency domain [21]. ReHo may be more sensitive to regional abnormalities than ALFF. Complementarily, ALFF may measure local spontaneous activity [27]. In the present study, we use the 2 indices of low frequency fluctuations of rsfMRI (ReHo, ALFF) to differ the two kinds of body constitution. Our present results demonstrated that ReHo analysis did not reveal the significant neural-behavior correlation of WHOQOL-BREF/BCQ-subtypes in BCQ-G/BCQ-all groups, respectively, which inferred that ReHo might be more sensitive inter-regional coherence. Nevertheless, ALFF may depict a significant negative association with WHOQOL-BREF/BCQ-subtypes (Table 4 and Table 5), which implied that ALFF may detect alteration of local spontaneous activity.

The current study has several limitations. First, we used a cross-sectional method, and the participants were scanned, and BCQ type was assessed for the first time. It is possible that BCQ type may change in different timings and environments. Second, the relatively small sample size in the present study may have diminished the ability to identify more neural activity changes. Third, there were inconsistent methods for classifying BC (for example, CCMQ and BCQ), which limits the reproducibility of our results. Finally, it is unknown whether the presently observed ReHo/ALFF alterations are associated with any subsequent development of pathological conditions. Longitudinal behavior assessments and neuroimaging studies of individuals with the BCQ-D phenotype are required to elucidate these results.

## 5. Conclusions

In conclusion, our combined ReHo and ALFF analyses revealed a significant decrease and increase in spontaneous brain activity, respectively, in various brain regions before structural changes in healthy adults of BCQ deficiency type. The BC subtype analysis showed that BCQ-G participants had consistent neural coherence in ReHo; however, BCQ-D individuals had attenuated neural activity in ALFF. This aberrant neural activity in the brains of BCQ-D individuals indicated an adaptive response to the QOL of these individuals. Future directions relating the BCQ deficiency type to psychological/physiological abnormalities should investigate the underlying mechanisms associated with changes in spontaneous activity in the resting brain state and recognize the importance of BC in individualizing the treatment strategies in TCM. 

## Figures and Tables

**Table 1:** Demographic data in different body constitution questionnaire-deficiency (BCQ) groups.

Characteristics	BCQ-G (*n* = 18)	BCQ-D (*n* = 14)	*p*-Value
Gender (M/F)	9/9	6/8	0.688
Age (years)	52.0 ± 6.32	46.36 ± 9.95	0.396
BMI	23.68 ± 2.39	24.04 ± 4.56	0.794
BCQ Subtype			
YinD	25.78 ± 2.92	34.64 ± 5.02	0.000 **
YaD	25.28 ± 2.65	37.21 ± 4.95	0.000 **
Phl & STA	20.06 ± 3.03	27.21 ± 4.84	0.000 **
WHOQOL-BREF, total	61.38 ± 8.28	58.25 ± 7.24	0.27
Physical	16.10 ± 1.82	15.51 ± 1.52	0.341
Psychological	14.78 ± 2.22	13.71 ± 2.33	0.199
Social	15.26 ± 2.30	14.38 ± 3.10	0.390
Environmental	15.25 ± 2.35	14.64 ± 1.20	0.386

Data are mean ± SD and were compared using the χ^2^ test (categorical variables) and independent *t*-test (continuous variables). BCQ-G, body constitution questionnaire-gentleness type; BCQ-D, body constitution questionnaire-deficiency type; BMI, body mass index; YinD, yin-deficiency; YaD, yang-deficiency; Phl & STA, phlegm and stasis; WHOQOL-BREF, World Health Organization Quality of Life Instruments (brief edition). ** *p* < 0.01.

**Table 2:** Pearson correlation matrix for the scales between WHOQOL-BREF and BCQ subtypes in different groups.

		All Healthy Adult Subjects (*n* = 32)					BCQ-Gentleness (*n* = 18)					BCQ-Deficiency (*n* = 14)				
		WHOQOL-BREF (Subdomains)					WHOQOL-BREF (Subdomains)					WHOQOL-BREF (Subdomains)				
		Phy	Psy	Soc	Evn	Sum	Phy	Psy	Soc	Evn	Sum	Phy	Psy	Soc	Evn	Sum
YinD	Pearson *r*	−0.226	−0.345	−0.294	−0.256	−0.317	−0.306	−0.395	−0.323	−0.206	−0.333	−0.019	−0.185	−0.244	−0.310	−0.220
	*p* value	0.214	0.53	0.102	0.158	0.077	0.217	0.105	0.191	0.412	0.177	0.949	0.528	0.400	0.280	0.451
YaD	Pearson *r*	−0.246	−0.430 *	−0.313	−0.277	−0.358 *	−0.243	−0.465	−0.413	−0.239	−0.375	−0.172	−0.469	−0.316	−0.453	−0.398
	*p* value	0.174	0.014 *	0.081	0.125	0.044 *	0.332	0.052	0.089	0.340	0.125	0.556	0.091	0.270	0.104	0.159
P&S	Pearson *r*	−0.165	−0.412 *	−0.241	−0.208	−0.293	0.034	−0.294	−0.274	−0.158	−0.202	−0.170	−0.416	−0.125	−0.158	−0.250
	*p* value	0.368	0.019 *	0.183	0.254	0.104	0.893	0.236	0.271	0.531	0.421	0.561	0.139	0.669	0.589	0.389

BCQ, body constitution questionnaire; YinD, yin-deficiency; YaD, yang-deficiency; P&S, phlegm and stasis; WHOQOL-BREF, World Health Organization Quality of Life Instruments (brief edition); Phy, physical domain of WHOQOL-BREF; Psy, psychological domain of WHOQOL-BREF; Soc, social domain of WHOQOL-BREF; Evn, environmental domain of WHOQOL-BREF; * *p* < 0.05 (2-tailed); *r*, Pearson correlation coefficients.

**Table 3:** Peak Montreal Neurological Institute (MNI) coordinates for regions exhibiting significant regional homogeneity (ReHo)/amplitude of low-frequency fluctuation (ALFF) values between the different body constitution groups.

Contrast	Region	BA	Size	*t* Score	Peak Coordinate		
					x	y	z
ReHo							
Within-group **							
BCQ-Gentleness							
Gentleness > 0	PCC, Left	23	16,838	33.85	−3	−48	24
Gentleness < 0	NS						
BCQ-Deficiency							
Deficiency > 0	PCC, Left	23	8516	30.36	−3	−54	30
	OFC, Right	11	4593	21.54	42	52	−12
Deficiency < 0	NS						
Between-group *							
Gentleness > Deficiency	Postcentral gyrus	2	69	5.37	51	−24	45
Gentleness < Deficiency	NS						
ALFF							
Within-group **							
BCQ-Gentleness							
Gentleness > 0	PCC	23	11639	17.34	−3	−54	21
Gentleness < 0	NS						
BCQ-Deficiency							
Deficiency > 0	OFC, Left	11	161	6.79	−33	33	−18
Deficiency < 0	Precentral gyrus, Left	6	29,955	52.63	−18	−18	54
Between-group *							
Gentleness > Deficiency	NS						
Gentleness < Deficiency	Pons/Cerebellum, Left		85	−3.86	−12	−36	−54

Peak coordinates refer to the Montreal Neurological Institute (MNI) space. Within-group one sample *t*-test, significance ** was set at the voxel level *p* < 0.001, followed by the family-wise error rate-corrected cluster level *p* < 0.05. Between-group 2 sample-*t*-test, significance * was set at the voxel level *p* < 0.005 followed by the family-wise error rate-corrected cluster level *p* < 0.05. ReHo, regional homogeneity; ALFF, the amplitude of low-frequency fluctuation; BA, Brodmann’s area; MNI, Montreal Neurological Institute; BCQ, Body Constitution Questionnaire; NS, nonsignificant; PCC, posterior cingulate cortex; OFC, orbitofrontal cortex.

**Table 4:** ReHo and ALFF values vary with WHOQOL-BREF scores in healthy adults in the BCQ-D group (*n* = 14).

Values	Region	BA	Size	*t*	Peak Coordinate		
					x	y	z
WHOQOL-BREF							
ReHo							
Physiological							
Positive	Cerebellum/lingual gyrus, R	19	77	6.99	12	−57	−6
Negative	Supramarginal gyrus, R	40	68	8.58	60	−21	33
ALFF							
WHOQOL-total							
Positive	NS						
Negative	Brainstem, R		67	7.84	6	−21	−24
Social							
Positive	NS						
Negative	vmPFC, L	10	168	5.75	−15	57	−6

Peak coordinates refer to the MNI space. Correlation analysis: Significance was set at the voxel level *p* < 0.005 followed by the family-wise error rate-corrected cluster level *p* < 0.05; ReHo, regional homogeneity; ALFF, amplitude of low-frequency fluctuation; WHOQOL-BREF, World Health Organization Quality of Life Instruments (brief edition); BCQ-D, body constitution questionnaire-deficiency type; BA, Brodmann’s area; MNI, Montreal Neurological Institute; vmPFC, ventromedial prefrontal cortex; NS, nonsignificant; R, right; L, left.

**Table 5:** ReHo and ALFF values vary with BCQ subscores in all healthy adults (*n* = 32).

Values	Region	BA	Size	*t*	Peak Coordinate		
					x	y	z
ReHo							
Yang-deficiency							
Positive	NS						
Negative	NS						
Yin-deficiency							
Positive	NS						
Negative	NS						
Phl & STA							
Positive	NS						
Negative	NS						
ALFF							
Yang-deficiency							
Positive	Parahippocampus, L	36	289	4.32	−21	−18	−24
Negative	NS						
Yin-deficiency							
Positive	NS						
Negative	NS						
Phl & STA							
Positive	Parahippocampus, L	36	207	5.05	−24	−6	−42
	Orbitofrontal cortex, L	11	304	4.52	−3	33	−15
Negative	NS						

Peak coordinates refer to the MNI space. Correlation analysis: Significance was set at the voxel level *p* Peak coordinates refer to the MNI space. Correlation analysis: Significance was set at the voxel level < 0.005 followed by the family-wise error rate-corrected cluster level *p* < 0.05; ReHo, regional homogeneity; ALFF, amplitude of low-frequency fluctuation; BCQ, body constitution questionnaire; Phl & STA, phlegm and stasis; BA, Brodmann’s area; NS, nonsignificant; R, right; L, left.

## Supplementary Materials

The following are available online at https://www.mdpi.com/2077-0383/8/7/951/s1, Figure S1: The flow chart of the study design, Figure S2: The correlation between ALFF and BCQ-subtypes.
