# Supplementary material for: Differing Spontaneous Brain Activity in Healthy Adults with Two Different Body Constitutions: A Resting-State Functional Magnetic Resonance Imaging Study
Source: J Clin Med. 2019 Jun 30;8(7):951. doi: 10.3390/jcm8070951 (PMC6678373; doi:10.3390/jcm8070951)
Supplement: Supplementary file 1 [file jcm-08-00951-s001.zip › FIGURE LEGENDS_S1-2.docx]

**FIGURE LEGEND**

**Figure S1.** The ﬂow chart of the study design.

M, male; F, female; rsfMRI, resting-state functional magnetic resonance imaging; WHOQOL-BREF, World Health Organization Quality of Life Instruments (brief edition); BCQ, Body Constitution Questionnaire.

**Figure S2** Correlation between ALFF and BCQ-subtypes

A: Positive correlations between the ALFF and Yan-deficiency in left parahippocampus 1(blue). B: Positive correlations between the between ALFF and Phl&STA in the left parahippocampus 2(blue). C: Positive correlations between the between ALFF and Phl&STA in the left OFC. (*P* < 0.05, FWE corrected). BCQ-D, Deficiency type of Body Constitution Questionnaire; ALFF, amplitude of low-frequency ﬂuctuation; FWE, family-wise error; Color scale denotes the t value. x, y, z, Montreal Neurological Institute coordinates; R, right; L, left.
