# Supplementary material for: Differing Spontaneous Brain Activity in Healthy Adults with Two Different Body Constitutions: A Resting-State Functional Magnetic Resonance Imaging Study
Source: J Clin Med. 2019 Jun 30;8(7):951. doi: 10.3390/jcm8070951 (PMC6678373; doi:10.3390/jcm8070951)
Supplement: Supplementary file 1 [file jcm-08-00951-s001.zip › Figure_S1.docx]

**Figure S1.** The ﬂow chart of the study design.

Healthy participants

2 excluded 2

1 tumor, 1 vascular anomaly

BCQ type: Gentleness

n = 18

Final recruited participants (n = 32) (16M, 16F)

Complete Scans: 34

rsfMRI (n = 35)

Failed scan (n = 1)

Recruited

16M /19F

BCQ type: deficiency

n = 14

Group divided according BCQ classification

Completed WHOQOL-BREF/BCQ
