# Supplementary material for: Differing Spontaneous Brain Activity in Healthy Adults with Two Different Body Constitutions: A Resting-State Functional Magnetic Resonance Imaging Study
Source: J Clin Med. 2019 Jun 30;8(7):951. doi: 10.3390/jcm8070951 (PMC6678373; doi:10.3390/jcm8070951)
Supplement: Supplementary file 1 [file jcm-08-00951-s001.zip › Figure_S1_legend.pdf]

Figure S1. The flow chart of the study design.

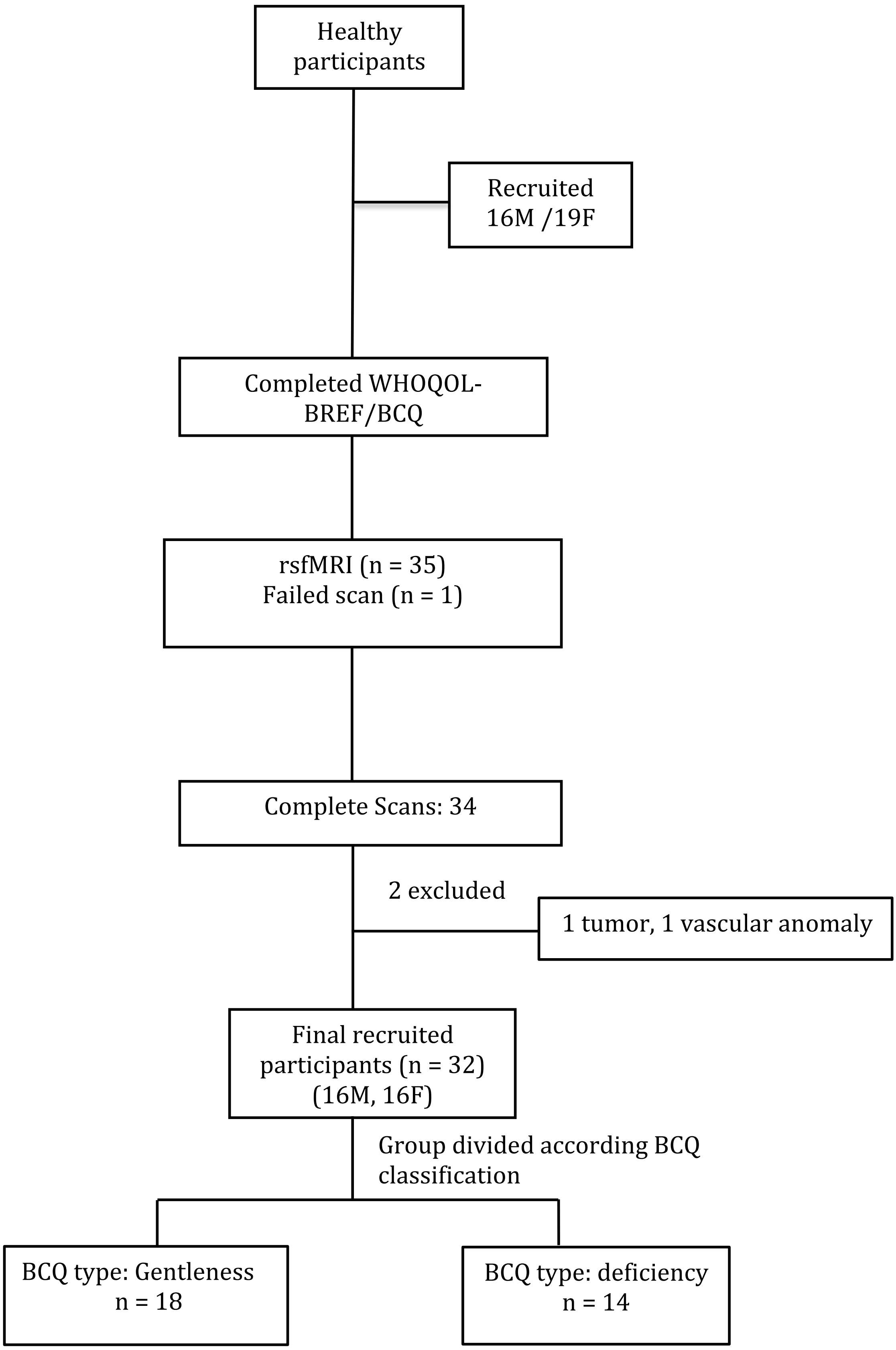

M, male; F, female; rsfMRI, resting-state functional magnetic resonance imaging; WHOQOL-BREF, World Health Organization Quality of Life Instruments (brief edition); BCQ, Body Constitution Questionnaire.
