# Supplementary figures and images for: Differing Spontaneous Brain Activity in Healthy Adults with Two Different Body Constitutions: A Resting-State Functional Magnetic Resonance Imaging Study
Source: J Clin Med. 2019 Jun 30;8(7):951. doi: 10.3390/jcm8070951 (PMC6678373; doi:10.3390/jcm8070951)

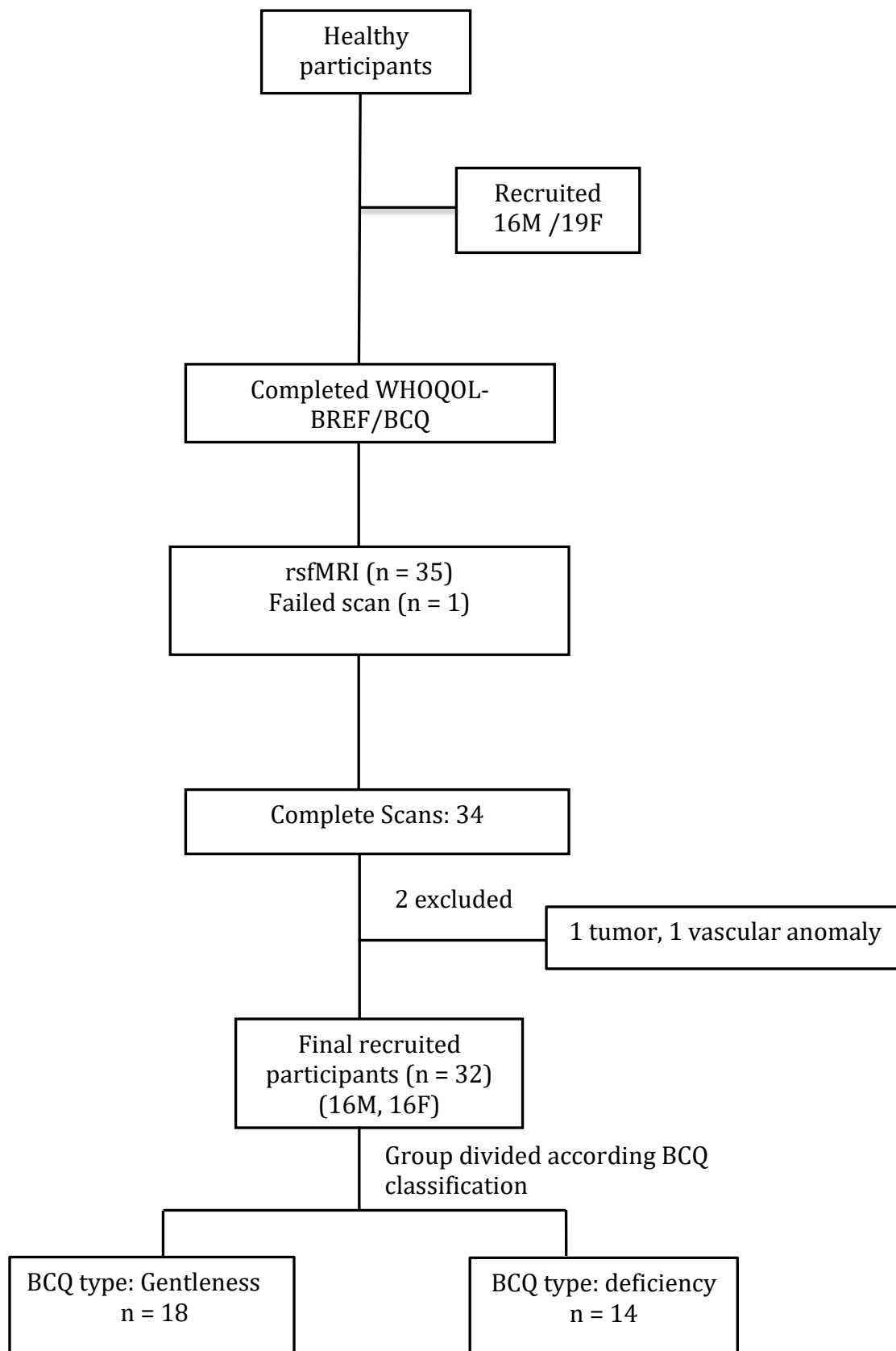

Supplement: Supplementary file 1 [file jcm-08-00951-s001.zip › Figure_S1.pdf]

Figure S2


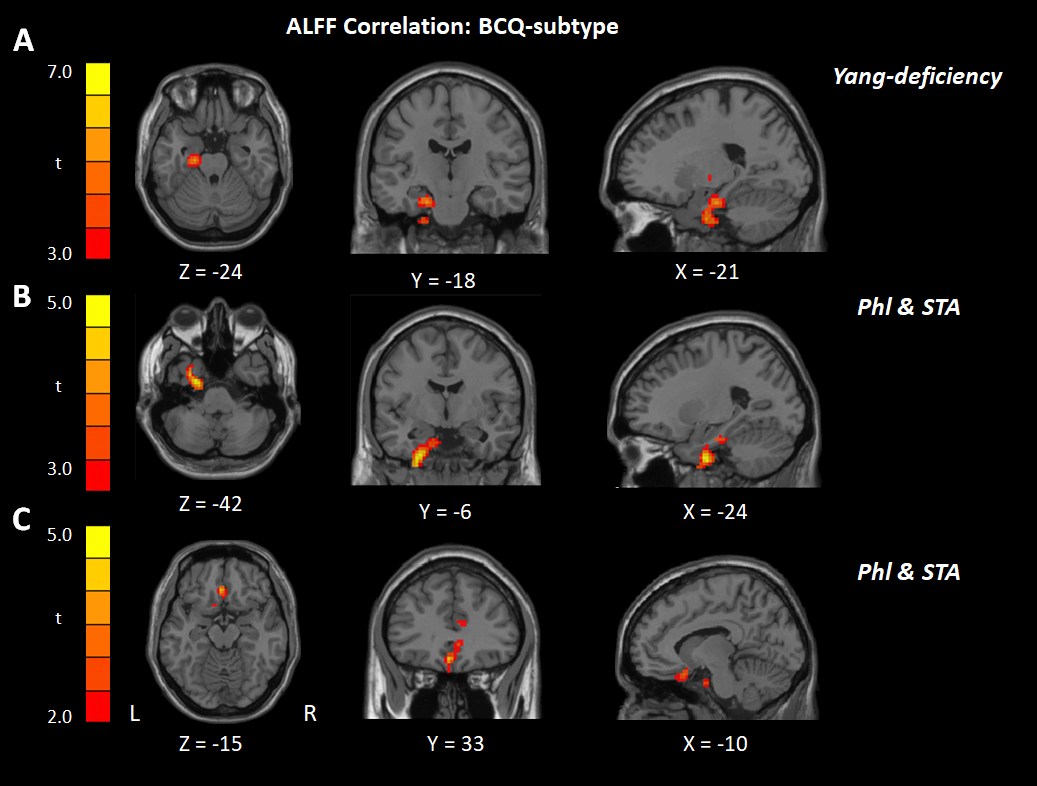

Supplement: Supplementary file 1 [file jcm-08-00951-s001.zip › Figure_S2.docx]
